# Endogenously-expressed NH_2_-terminus of circumsporozoite protein interferes with sporozoite invasion of mosquito salivary glands

**DOI:** 10.1186/s12936-016-1207-8

**Published:** 2016-03-10

**Authors:** Bianca B. Kojin, André Luis Costa-da-Silva, Ceres Maciel, Dayane Alves Henriques, Danilo O. Carvalho, Kelcie Martin, Osvaldo Marinotti, Anthony A. James, Myrna C. Bonaldo, Margareth Lara Capurro

**Affiliations:** Laboratório de Mosquitos Geneticamente Modificados, Departamento de Parasitologia, Instituto de Ciências Biomédicas, Universidade de São Paulo, São Paulo, SP 05508-000 Brazil; BSL3+ Laboratory, Instituto de Ciências Biomédicas, Universidade de São Paulo, São Paulo, SP 05508-900 Brazil; Department of Molecular Biology and Biochemistry, University of California Irvine, Irvine, CA 92697 USA; Department of Microbiology and Molecular Genetics, University of California, Irvine, CA 92697 USA; Laboratório de Biologia Molecular de Flavivirus, Instituto Oswaldo Cruz/FIOCRUZ, Manguinhos, RJ 21040-360 Brazil

**Keywords:** *Plasmodium gallinaceum*, *Plasmodium falciparum*, Circumsporozoite protein, Sindbis virus, Transgenic mosquitoes, *Aedes aegypti*

## Abstract

**Background:**

The circumsporozoite protein is the most abundant polypeptide expressed by sporozoites, the malaria parasite stage capable of infecting humans. Sporozoite invasion of mosquito salivary glands prior to transmission is likely mediated by a receptor/ligand-like interaction of the parasites with the target tissues, and the amino (NH_2_)-terminal portion of CSP is involved in this interaction but not the TSR region on the carboxyl (C)-terminus. Peptides based on the NH_2_-terminal domain could compete with the parasites for the salivary gland receptors and thus inhibit penetration.

**Methods:**

Peptides based on the NH_2_-terminus and TSR domains of the CSP from avian or human malaria parasites, *Plasmodium gallinaceum* and *Plasmodium falciparum*, respectively, were expressed endogenously in mosquito haemolymph using a transient (Sindbis virus-mediated) or stable (*piggyBac*-mediated transgenesis) system.

**Results:**

Transient endogenous expression of partial NH_2_-terminus peptide from *P. falciparum* CSP in *P. gallinaceum*-infected *Aedes aegypti* resulted in a reduced number of sporozoites in the salivary glands. When a transgenic approach was used to express a partial CSP NH_2_-terminal domain from *P. gallinaceum* the number of sporozoites in the salivary glands did not show a difference when compared to controls. However, a significant difference could be observed when mosquitoes with a lower infection were analysed. The same result could not be observed with mosquitoes endogenously expressing peptides based on the TSR domain from either *P. gallinaceum* or *P. falciparum.*

**Conclusion:**

These results support the conclusion that CSP partial NH_2_-terminal domain can be endogenously expressed to promote a competition for the receptor used by sporozoites to invade salivary glands, and they could be used to block this interaction and reduce parasite transmission. The same effect cannot be obtained with peptides based on the TSR domain.

**Electronic supplementary material:**

The online version of this article (doi:10.1186/s12936-016-1207-8) contains supplementary material, which is available to authorized users.

## Background

Malaria parasites have a complex developmental cycle in both the vertebrate and vector mosquito hosts. The sporozoites are the most versatile invasive stage of the parasite and are unique in having the ability to invade two types of cells, their primary targets in their vertebrate hosts and those of the mosquito salivary glands [1]. Furthermore, target-cell specificity supports the hypothesis that the localization and penetration of mosquito salivary glands by sporozoites is mediated by receptors [2–5]. Two mosquito proteins, circumsporozoite binding protein (CSPBP) and saglin, were identified as receptors for sporozoites and these interact with the parasite-expressed circumsporozoite protein (CSP) and thrombospondin-related anonymous protein (TRAP), respectively [4, 5]. Merozoite apical erythrocyte-binding ligand (MAEBL) is another proposed sporozoite ligand involved in attachment and invasion of mosquito salivary glands [6], however, no receptor has been characterized yet.

CSP is the predominant sporozoite surface antigen and important for mediating recognition and invasion of the salivary glands (as reviewed [7, 8]). It is expressed by a single copy gene [9], and has a large central immuno-dominant domain comprising tandem repeats of small peptides. The amino-(NH_2_) terminus comprises a domain of hydrophobic amino acid that makes up the secretory signal peptide, and a pentapeptide (KLKQP) designated Region I, that contains a proteolytic cleavage site. Cleavage is required for vertebrate hepatocyte cell invasion, but is not necessary for salivary gland infection in the mosquito host [10, 11]. Furthermore, the NH_2_-terminus together with the repeat domain is responsible for sporozoite development and *Plasmodium berghei* mutants lacking these regions do not produce free sporozoites [12]. The carboxyl-(C) terminus, which has sequence similarity to the thrombospondin type-1 repeat (TSR) superfamily, has an 18-amino acid sequence (EWSXCXVTCGXG(V/I)XXRX(K/R) designated Region II [13–15], and a putative glycosylphosphatidylinositol (GPI) anchor attachment site. Both Regions I and II are conserved highly among *Plasmodium* species [16].

*Plasmodium falciparum* CSP binds with greater avidity to the medial lobe and distal portions of the lateral lobes of *Anopheles stephensi* salivary glands than any other mosquito organs that are in contact with the haemolymph, and these lobes are invaded preferentially by sporozoites [17]. A synthetic peptide encompassing Region I, plus the additional five amino acids immediately adjacent to the amino-end of the endogenous sequence, inhibits CSP binding to salivary glands [17]. In addition, synthetic peptides encompassing *P. falciparum* Region I inhibit by 80 % sporozoite invasion of salivary glands [18]. Mutant sporozoites expressing a CSP in which Region I is deleted invade salivary glands with a 10–15 % lower efficiency compared with controls, and mutants with a complete N-terminal deletion have ten-fold fewer salivary gland sporozoites compared with controls [11]. In contrast, *P. berghei* mutant sporozoites expressing a *P. falciparum* CSP lacking Region I showed no impairment of motility or infectivity in the vector host, however, disruption of Region II interfered with motility and impaired sporozoite invasion of salivary gland [19].

Transient (recombinant Sindbis virus) and stable (*piggyBac*-mediated mosquito transformation) expression systems were used to study competition between different peptides from *Plasmodium gallinaceum* and *P. falciparum* based on NH_2_-terminus or TSR domains of CSP and conspecific sporozoites for receptors in the salivary gland of *Aedes aegypti*. The data presented here shows that only NH_2_-terminal peptides interfere with sporozoite penetration of mosquito salivary glands. Furthermore, a heterologous *P. falciparum* CSP partial NH_2_-terminus peptide also can block *P. gallinaceum* sporozoite invasion of the *Ae. aegypti* salivary glands. These results support the conclusion that the partial NH_2_-terminus peptides from both *P. gallinaceum* and *P. falciparum* may act as ligands in the invasion of mosquito salivary glands, and the possibility of using the peptides to prevent parasite transmission.

## Methods

### Mosquitoes and maintenance of *Plasmodium gallinaceum* life cycle

The *Ae. aegypti* Higgs white-eyed strain [20] was maintained in the insectary at Universidade de São Paulo, Parasitology Department at 27 ± 2 °C, 80 % relative humidity, 12/12 h day/night, with access ad libitum to a 10 % sucrose solution in water. Anesthetized mice were used when required for blood feeding. Larvae were fed with powdered fish food (Tetramin, Blacksburg, VA, USA). *P. gallinaceum* were maintained as described [21]. Mosquitoes were fed on chickens infected with *P. gallinaceum* (strain 8A, obtained from A. Krettli, René Rachou Institute of Research, FIOCRUZ, MG, Brazil). Infection was determined by the presence of sporozoites in the salivary glands and infected females were fed thereafter on uninfected chickens.

### Sindbis virus cloning and infectious virus production

**Fig. 1:**
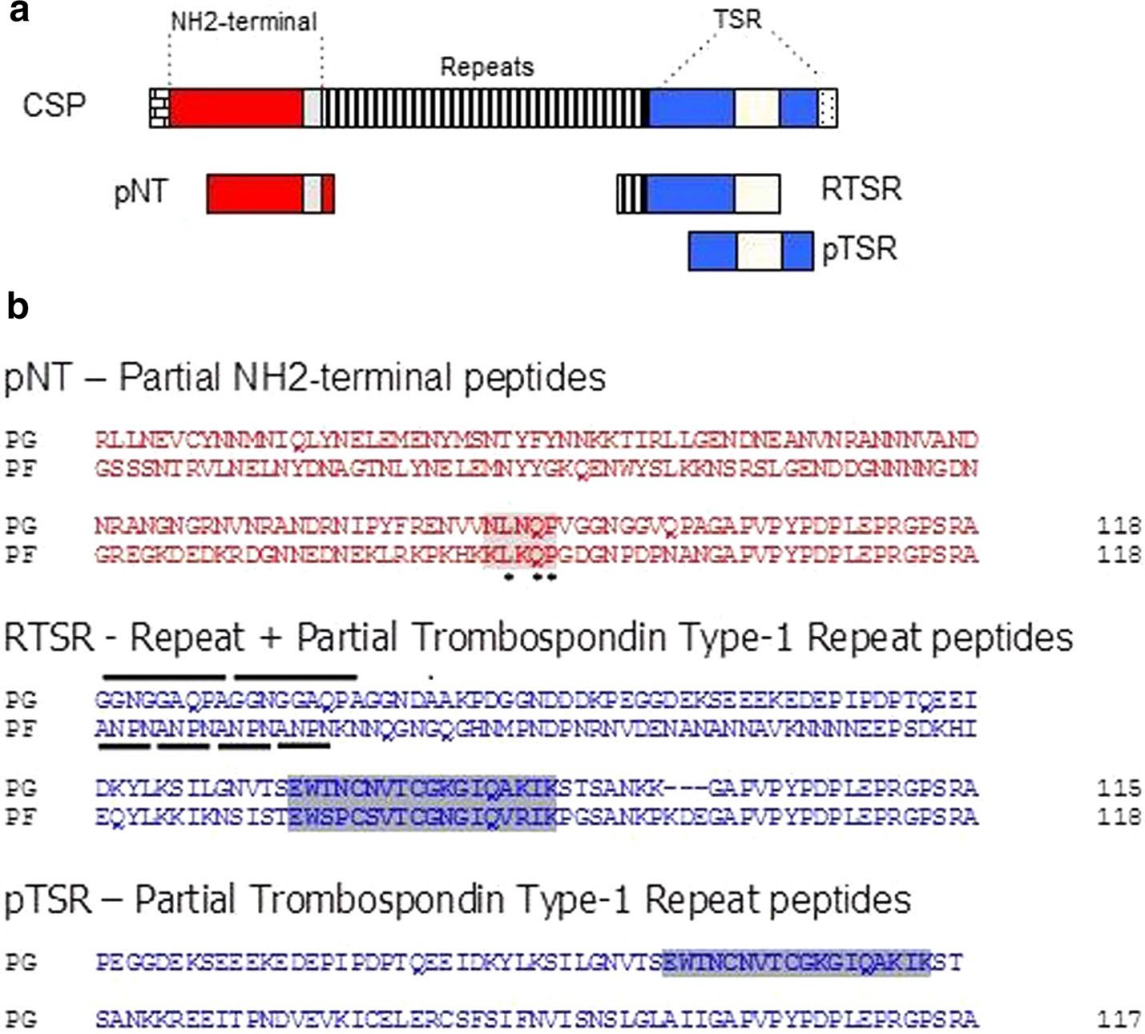
Partial NH_2_-terminus and partial TSR peptides endogenously expressed from *Plasmodium gallinaceum* and *Plasmodium falciparum.* **a** Schematic representation of circumsporozoite protein and endogenously-expressed peptides. The *open bricked block* represents the signal peptide; the *red block* represents the NH_2_-terminus; the *light gray block* represents the conserved motif Region I; the *striped block* represents the tandem repeats; the *blue block* represents the TSR domain; the *gray block* represent the region II motif and the *dotted block* the putative GPI anchor. **b** Alignment of amino acid sequences of endogenously-expressed peptides from *P. gallinaceum* (PG) and *P. falciparum* (PF) CSPs. Key: *red*, partial NH_2_-terminal; *light gray*, Region I; *asterisks*, conserved amino acids; *underlined*, tandem repeats; *blue*, partial TSR; and *dark gray*, Region II

Peptides designated pNH_2_-T, RTSR and pTSR, encompass NH_2_-terminus portion of CSP, the repeat plus a partial portion of TSR domain and only a partial portion of TSR domain, respectively (Fig. 1). The prefix ‘Pg’ or ‘Pf’ is added before each peptide to differentiate *P. gallinaceum* from *P. falciparum* CSP, respectively. The gene amplification (PCR) of the coding sequences of the recombinant peptides was done in two steps so as to include a region 72 base-pairs (bp) in length encoding the *Maltase*-*like* I signal peptide (to direct recombinant peptides to mosquito haemolymph) [22] at the 5′-end of the fragments. In the first PCR, primers specific for each fragment added the 3′-end of the *Maltase*-*like* I signal peptide, followed by a second PCR with the general primer pair to complete the *Maltase*-*like I* signal peptide 5′-end with *XbaI/ApaI* and *XbaI* restriction enzyme site sequences for directional cloning. All primer sequences used to build the constructs listed in Additional file 1.

After the two-step amplification, the fragments were cloned into *p*GEM-T (Promega) and *XbaI* digestion was used to recover the final constructs, which then were sub-cloned into the Sindbis virus expression plasmid, TE/3′2 J later (Additional file 2), generating Sin-PgpNT and Sin-PfpNT representing the peptides encompassing NH_2_-terminus CSP of *P. gallinaceum* and *P. falciparum*, respectively, and Sin-PgRTSR (*P. gallinaceum*) and Sin-PfRTSR and Sin-PgpTSR (*P. falciparum*) representing peptides encompassing the TSR domain of CSP.

Plasmids containing Sin-PgpNT, Sin-PfpNT, Sin-PgRTSR, Sin-PfRTSR, or Sin-PgpTSR were linearized with *XhoI* and transcription in vitro was performed with AmpliScribe™ T7-Fash™ (Epicentre) kit. Transcribed RNA was transfected with Lipofectamine™ Reagent (Invitrogen) in BHK-21 cells, viruses were transferred to C6/36 cells 72 h later, harvested at four days post-infection and stored in −80 °C until used. Aliquots of viruses were titrated using C6/36 cells through an end-point assay [23].

### Transgene assembly and *Aedes aegypti* genetic transformation

A PgpNT fragment was amplified with primers designed to add the *BglII* and *NotI* restriction sites for directional cloning into the vector pSLfa [AeVg] [24]. This plasmid has the *Ae. aegypti Vitellogenin I* (*VgI*) gene promoter for blood-meal induced expression of the peptides in the haemolymph [24]. The FOW_*BglII* and REV_*NotI* primers (Additional file 1) were used to amplify these fragments from the Sin-PgpNT plasmid. After amplification, the product was cloned first into the TOPO TA cloning (Invitrogen), sequenced and sub-cloned into the pSLfa[AeVg] plasmid. The cassette [AeVg-PgpNT] was excised using *AscI* and sub-cloned into pBac 3XP3-eGFPafm. The resulting vector generated is designated pBac[3XP3-eGFP-AeVg-PgpNT] (Additional file 2). Transgenic *Ae. aegypti* lines were generated by injecting preblastoderm embryos with a mixture of the donor plasmid pBac[3XP3-eGFP-AeVg-PgpNT] (300 µg/µL) and the pBac helper plasmid (200 µg/µL) using procedures described previously [25].

### RT-PCR

Whole-body total RNA was isolated from Sindbis virus-infected and transgenic *Ae. aegypti* with TRIzol (Invitrogen) and treated with DNAse I (Invitrogen). The amplification of diagnostic products was done using the OneStep RT-PCR kit (Qiagen) and primers listed in Additional File 1. The reaction mixture was incubated at 50 °C for 30 min and 95 °C for 15 min. Amplification conditions were 94 °C for 1 min followed by 30 cycles of 94 °C for 1 min, 60 °C for 1 min and 72 °C for 1 min and a final step of 10 min at 72 °C.

### Salivary gland infection assay in dsSindbis-infected *Aedes aegypti*

Four day-old adult females were injected with 0.5 μL of a solution containing 10^6^ 50 % tissue culture infective dose (TCID_50 %_) Sin-PfpNT, Sin-PgRTSR, Sin-PfRTSR and Sin-PgpTSR and control Sin-EGFP (recombinant Sindbis virus expressing EGFP) infectious virus. After 3 days, mosquitoes were allowed to feed on *P. gallinaceum*-infected chickens (8–11 % parasitemia) and only fully-engorged females were used in the experiments. Salivary glands pairs were dissected on the 8th day after the infectious blood meal according to *P. gallinaceum* infection profile [26] (corresponding to 11th day after dsSindbis infection) and individually homogenized in 10 µL of phosphate-buffered saline (PBS), placed in a haemocytometer and sporozoite numbers determined by phase-contrast microscopy.

### Salivary gland infection assay in transgenic *Aedes aegypti*

Transgenic and wild-type female *Ae. aegypti* were fed on *P. gallinaceum*-infected chickens with an 8–11 % parasitaemia. Five days after the first blood feeding, a second blood meal using healthy mice was offered to maintain the expression from the blood meal-induced *VgI* gene promoter driving the recombinant CSP peptides [27]. Sporozoite number was determined as described previously.

### Statistical analysis

The data were analysed with GraphPad InStat version 3.00 Software and Mann–Whitney and unpaired t test was used to assess the statistical significance of the differences between control and experimental groups, a *p* value of <0.05 was considered statistically significant.

## Results

### dsSindbis virus production in BHK21 cells and mRNA expression in dsSindbis virus infected mosquitoes

**Fig. 2:**
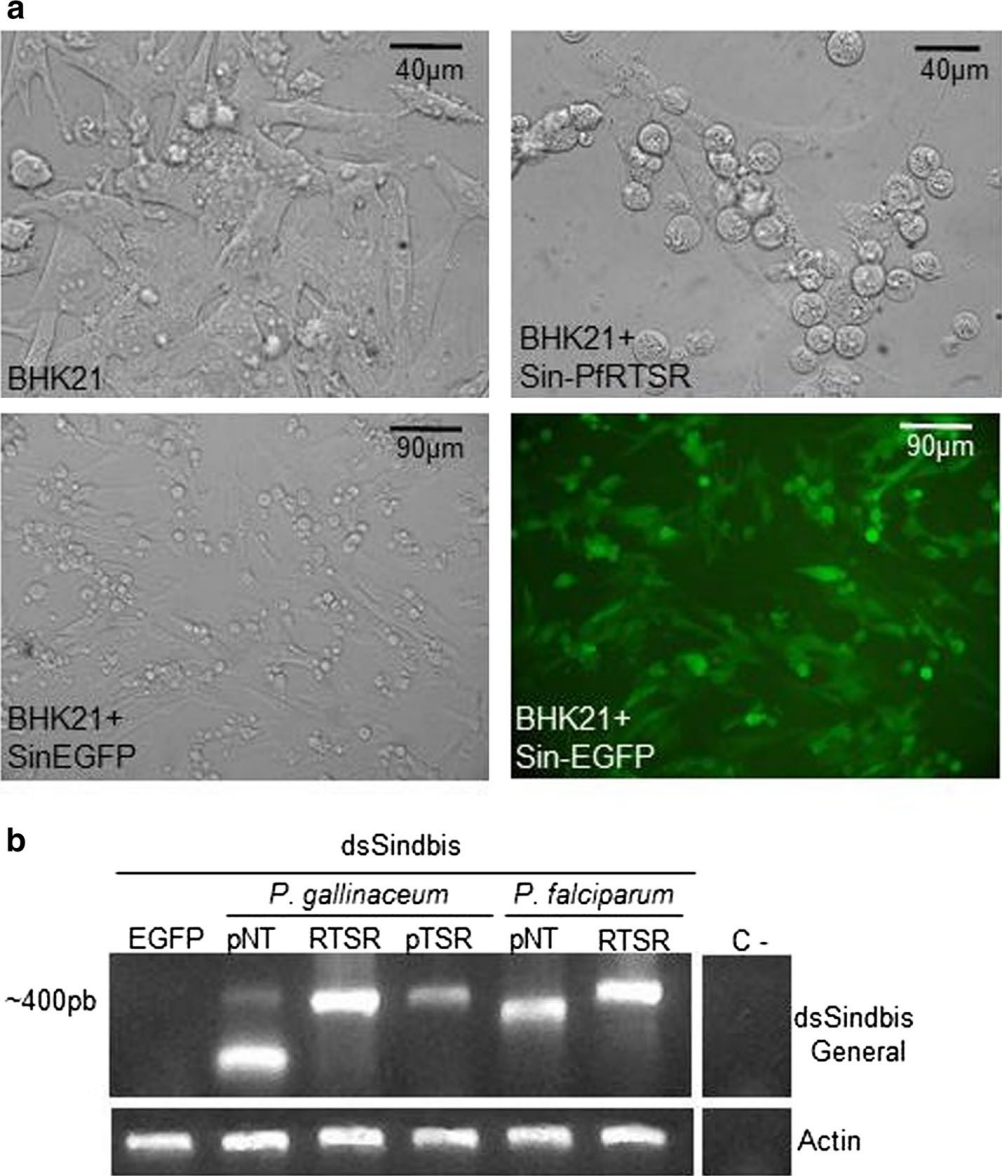
Sindbis virus production in BHK21 cells and mRNA expression in dsSindbis virus infected mosquito. **a** Light microscope observation of a BHK21 cell monolayer (*top left panel*), BHK21 stably transfected with Sin-PfRTSR (*top right panel*) or dsSin-EGFP (*bottom left panel*) and fluorescence microscopy of BHK-21 transfected with dsSin-EGFP (*bottom right panel*). **b** Detection of transcript from dsSindbis virus-infected mosquitoes. Total RNA samples were extracted from female mosquitoes eleven days after thoracic microinjection with each of the Sin-EGFP, Sin-PgpNT, Sin-PgRTSR, Sin-PgpTSR, Sin-PfpNT, Sin-PfRTSR viral particles, and used in a RT-PCR reaction. The same RNA samples were also analysed by actin-specific primers (Additional file 1). Abbreviations: *C* negative control (without template)

The infectivity of the experimental and control recombinant viruses was verified by the observation of cytopathic effects and EGFP expression in BHK-21 cells (Fig. 2a). Examination of control BHK-21 cells monolayers by phase contrast microscopy shows star-shaped cells with a fusiform appearance and a confluent monolayer of ~80 %. Cytopathic effects were recognized on BHK-21 monolayers 24 h after dsSindbis infection. Specifically, it was observed loss of cell structure (round cells) and detachment from surface of the flask. Also, fluorescence microscopy shows that the EGFP could be detected in most BHK-21 cells infected with Sindbis-GFP, with stronger signals in the round cells. RT-PCR analyses verified that females injected intrathoracically with Sin-PfpNT, Sin-PgRTSR, Sin-PfRTSR, Sin-PgpTSR and the control Sin-EGFP viral particles were accumulating the respective transcripts on the 11th day post-viral infection, which is the day the *P. gallinaceum* challenge was performed (Fig. 2b). Although Sin-PgpNT-infected mosquitoes showed the expected amplification product, the accumulated level of transcripts appeared lower than what was observed in the other virus-injected groups. Additionally, a smaller, unanticipated amplification product was detected in Sin-PgpNT-injected mosquitoes (Fig. 2b), thus PgpNT peptide expression was analysed using a transgenic approach.

### *Plasmodium gallinaceum* infection in *Aedes aegypti* transiently expressing CSP peptides

**Fig. 3:**
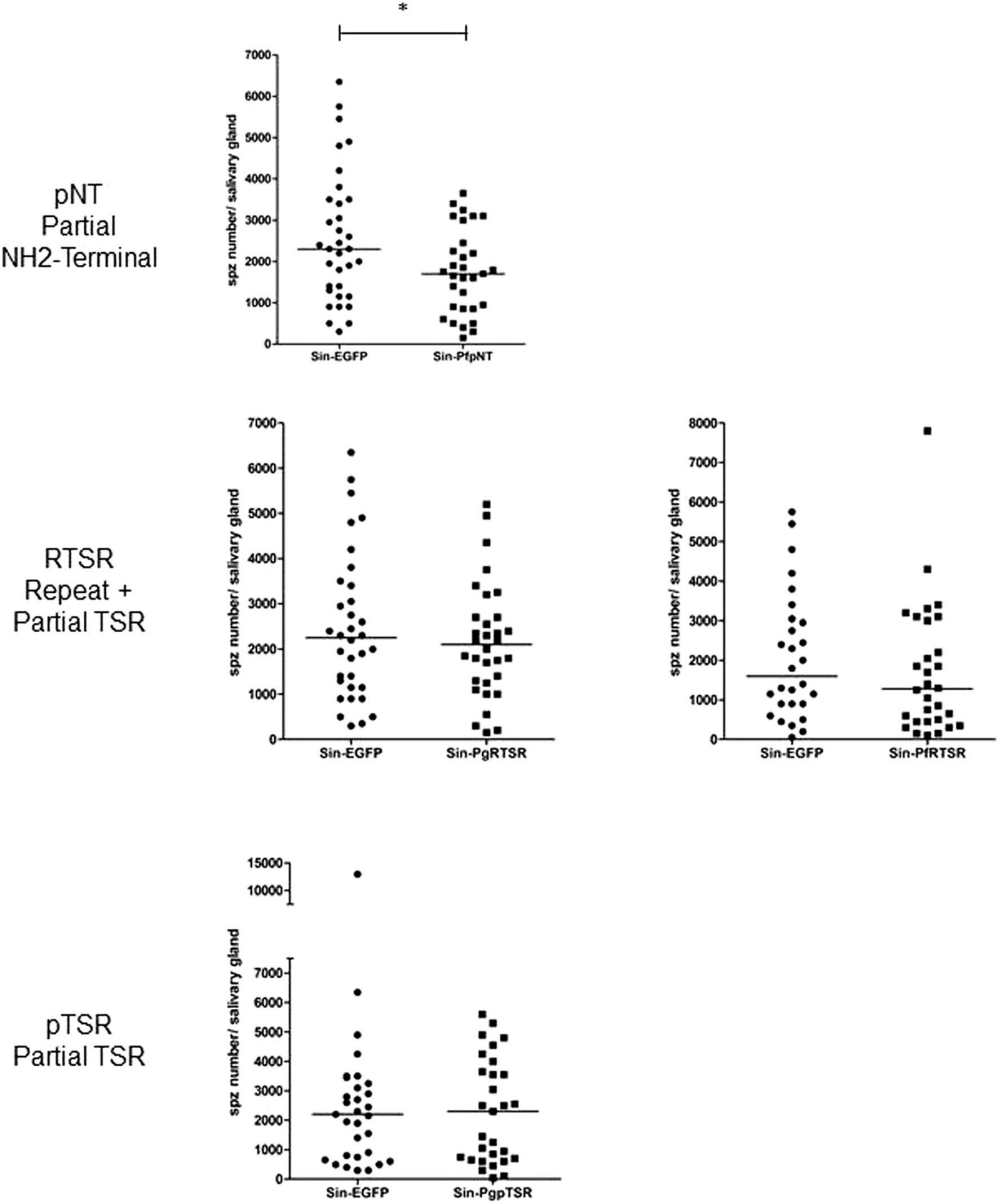
*Plasmodium gallinaceum* infection of individual dsSindbis-infected and control mosquitoes. *Aedes aegypti* females were injected intrathoracically with Sin-PfpNT, Sin-PgRTSR, Sin-PfRTSR, Sin-PfpTSR and control Sin-EGFP, and were fed three days later on *P. gallinaceum*-infected chickens. Salivary glands were dissected eight days after infected blood meal and sporozoite counted using phase-contrast microscopy. *Solid circles* (control Sin-EGFP) and *squares* (Sin-PfpNT, Sin-PgRTSR, Sin-PfRTSR, Sin-PfpTSR) represent individual mosquitoes with sporozoites detected in their salivary glands. The *horizontal bars* represent the median. A *p* value of <0.05 was considered statistically significant

Sindbis viruses were injected into mosquitoes to endogenously express the CSP peptides to promote competition for the salivary gland receptors. The numbers of sporozoites recovered from salivary glands of mosquitoes injected with Sin-PgRTSR, Sin-PfRTSR and Sin-PgpTSR viral particles were similar to those recovered from the control group injected with dsSin-EGFP, and no statistical differences were observed (*p* = 0.234, 0.111 and 0.355, respectively) (Fig. 3). However, expression of Sin-PfpNT in infected mosquitoes resulted in a 31.5 % reduction of mean sporozoites numbers when compared with controls (*p* = 0.018). The results demonstrate that the dsSindbis virus system is capable of expressing CSP derivative peptides, and that partial CSP NH_2_-terminal peptides from *P. falciparum* are capable of blocking *P. gallinaceum* infection of *Ae. aegypti* salivary glands while peptides based on TSR domains from either *P. gallinaceum* or *P. falciparum* are not (Figs. 2, 3). These data are consistent with competition between the peptides and sporozoites for a reception on the mosquito salivary glands.

### Analyses of transgenic *Aedes aegypti* endogenously expressing *Plasmodium gallinaceum* partial NH_2_-terminus CSP peptide

**Fig. 4:**
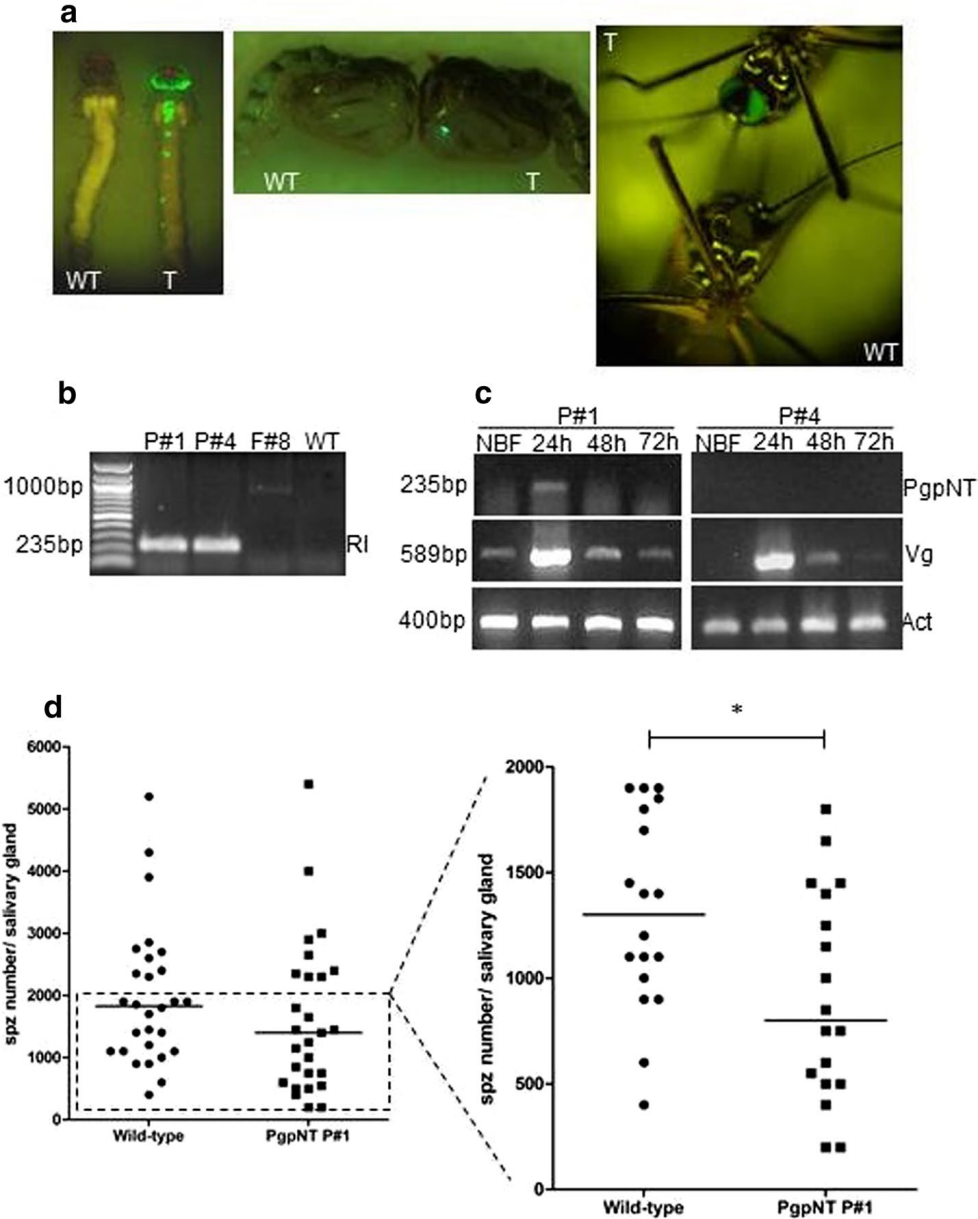
Generation, characterization and *Plasmodium gallinaceum* infections of pBac[3XP3-eGFP-AeVg-PgpNT] transgenic mosquitoes. **a** EGFP fluorescence in both eyes of larvae, pupae and adults of transgenic mosquitoes (T) but not in wild-type controls (WT). Only in larvae EGFP could be detected in the neural tube. **b** Genomic DNA was extracted from transgenic lines pBac-PgpNT P#1, P#4 and F#8 and used for PCR with specific primers for the PgpNT fragment. **c** Detection of transgenic transcript from pBac-PgpNT P#1 mosquitoes. Total RNA was extracted from non-blood fed (NBF), 24, 48 and 72 h post-blood meal females mosquitoes and used on a RT-PCR reaction with specific primers for PgpNT sequence. The same RNA samples were also analysed by *vitellogenin* (Vg) and *actin* (Act) gene-specific primers (Additional file 1). **d** Transgenic pBac-PgpNT P#1 and wild-type adult females were fed in *P. gallinaceum*-infected chickens, five days later a second blood meal with healthy mice was offered. Salivary glands were dissected eight days after infected blood meal and sporozoite counted using phase-contrast microscopy. *Solid circles* and *squares* represent individual wild-type and transgenic mosquitoes respectively, with sporozoites detected in their salivary glands. The *horizontal bars* represent the median, a *p* value of <0.05 was considered statistically significant

Since the PgpNT peptide could not be tested using the dsSindbis system, a transgenic approach was used to evaluate it. Three independent transgenic lines (P#1, P#4 and F#8) carrying pBac-PgpNT were recovered following injection of 1098 *Ae. aegypti* embryos, standard rearing and screening procedures. From the embryos microinjected, 111 larvae were recovered (10 % eclosion rate) but only 62 made it to adulthood and provided fertile families. From the progeny of these families, 3248 larvae were analysed and 77 were positive for EGFP, 10 for P#1, 66 for P#4 and 1 for F#8. All EGFP-positive larvae had fluorescence in their eyes and neural tubes but not in the anal papillae, and only in the eyes of pupae and adults (Fig. 4a).

Transgene insertions were confirmed by diagnostic gene amplification using primers specific for the PgpNT sequence (Additional file 1). The expected PgpNT fragments were amplified from P#1 and P#4 genomic DNA, but a fragment larger than expected was amplified from the F#8 line (Fig. 4b). RT-PCR on pools of 10 P#1 females verified transcript presence and accumulation of the PgpNT transgene product at 24 h post-blood meal (hPBM) but not in samples prepared from mosquitoes 48 and 72 h PBM. This is consistent with the expression profile of the endogenous *VgI* gene. No cDNA amplification was detected in the P#4 line (Fig. 4c). Both lines F#8 (no diagnostic gene fragment of the expected size) and P#4 (unexpected expression profile) were discarded and only P#1 was used in subsequent experiments.

### *Plasmodium gallinaceum* infection in *Aedes aegypti* pBac-PgpNT P#1 transgenic mosquitoes

In order to evaluate the capacity of PgpNT peptide to compete with sporozoites for salivary gland receptors, transgenic P#1 and wild-type *Ae. aegypti* females were fed on chickens with 8–11 % *P. gallinaceum* parasitaemia. The numbers of sporozoites recovered from salivary glands of challenged P#1 individual transgenic mosquitoes did not differ from the wild-type (*p* < 0.2097). Previous experiments showed that *P. gallinaceum* infection in *Ae. aegypti* (Higgs strain) provides a mean infection number of 2086 sporozoites per salivary gland (C. Maciel, unpublished data). Thus mosquitoes with numbers below that were considered as low infection. Restricting analysis to those mosquitoes a significant 30.3 % reduction (*p* = 0.0186) in parasites recovered from salivary glands was observed when compared with wild-type controls (Fig. 4). The same analysis was performed using the mosquitoes showing the infection level above the threshold established, and there is no significant difference between pBac-PgpNT line P#1 and control mosquitoes. Moreover, there is no significant difference if the same analysis is performed with the TSR-based peptides Sin-PgRTSR, Sin-PfRTSR, Sin-PfpTSR (Additional file 3), showing that this event only occurs for NH_2_-terminus peptides.

## Discussion

CSP represents 5–15 % of the total protein expressed in sporozoites and is highly immunogenic [28, 29]. These characteristics make CSP an excellent candidate for malaria vaccine development. RTS,S AS01, a recombinant protein candidate malaria vaccine that targets the *P. falciparum* CSP is the first to reach phase three clinical testing and is partially effective against clinical disease in young African children up to 4 years after vaccination, according to final trial data [30]. Other important properties of CSP include its ligand-like interaction with mosquito salivary gland receptors and this makes it a target for disrupting parasite invasion in this tissue and thus blocking transmission. The results presented here are the first to show that endogenous production of peptides encompassing NH_2_-terminus of CSP protein using either a dsSindbis virus system or transgenic approach, inhibit sporozoite penetration into salivary gland using *Ae. aegypti*–*P. gallinaceum* model. Moreover this is the first report of a heterologous effect of the *P. falciparum* NH_2_-terminus of CSP-peptide in the *P. gallinaceum* model.

Controversy accompanies the published conclusions of the roles of CSP Regions I and II in receptor-mediated salivary gland recognition [11, 17–19]. The results presented here support the conclusion that the NH_2_-terminus (containing Region I) has a role in salivary gland recognition but not TSR (with Region II) in *P. gallinaceum* infection in a model system. Previous reports of a role for Region II were based on experiments performed with *P. berghei* [19, 31], however, the genetically-ablated parasites used were disabled in their gliding ability, and this makes it difficult to distinguish between disruption of a receptor-ligand interaction or the lack of motility as a cause of the reduced salivary gland infections. It also is possible that a mutation in Region II inhibits parasites egression from oocysts even though they produce the same numbers of oocyst sporozoites [31]. Since the previous study did not measure the number of sporozoite in the haemocoel, it is possible that this is the cause of the impairment phenotype observed in both studies. Furthermore, once the NH_2_-terminus is removed from mutated parasites, the TSR domain is exposed, switching parasites from a migratory state to an adhesive state reducing the numbers of sporozoite capable of reaching and invading the salivary glands [11]. Thus, it is unlikely that the TSR + Region II is acting as a ligand for sporozoites for salivary gland invasion.

An evolutionarily-conserved role for the partial NH_2_-terminus portion of CSP in salivary gland invasion is supported here, since it was observed that not only peptides from *P. gallinaceum* but also from *P. falciparum* are able to disrupt *P. gallinaceum* sporozoites penetration of *Ae. aegypti* salivary glands. Also, *P. gallinaceum* and *P. falciparum* are thought to be more closely-related than *P. falciparum* with other human malaria parasites [32–35]. Thus, information obtained with *Ae. aegypti*–*P. gallinaceum* model studies may be relevant to *P. falciparum*.

Transient expression of Sindbis-PgpNT resulted in a lower mRNA production than the other dsSindbis constructions and this most likely resulted in reduced peptide production, which in turn would not be enough to interfere with sporozoite penetration of the salivary glands. Also, the non-specific amplification product was most likely due to virus instability in cell culture passages that could select viruses with different sequences or for those lacking the transgene [36].

The choice of the *VgI* promoter in the transgenesis construct was based on its high level of expression and peak induction time (~24 h PBM). Previous work has shown its effectiveness in expressing heterologous proteins in *Ae. aegypti* transgenic lines [24, 27]. The signal peptide from the *Ae. aegypti maltase*-*like I* gene also was added to direct recombinant peptides into mosquito haemolymph [22].

Although all the elements chosen to generate the transgenic line were tested in previous reports, transgenic lines P#2 and F#8 did not express the transgene and this could be related to position effects, a phenomenon frequently seen in mosquito transgenesis [37]. Furthermore, F#8 had an abnormal fragment when amplified with specific primers, and a recombination event might have occurred disrupting the integrity of the transgene, so subsequent experiments were performed with line P#1 that did not present these problems.

The initial analysis of parasite challenges of the pBac-PgpNT line P#1 did not reveal a statistically-significant difference for *P. gallinaceum* sporozoite in salivary glands between them and non-transgenic controls. Although this result first suggested the incapability of transgenic mosquitoes to block parasite entrance in the salivary gland, a more profound analysis of that initial outcome was performed. Since ligand/parasite competition should be interfered by parasite excess binding to the salivary gland, it was defined a threshold (<2086 sporozoites/salivary gland) to determine mosquitoes with low infection levels and a significant reduction in parasites recovered from salivary glands was observed between pBac-PgpNT line P#1 and control mosquitoes below the threshold, but not above (Additional file 3). Therefore, if the number of parasites is not in excess (saturation), it is possible to block the receptors thus inhibiting sporozoite salivary gland invasion, on the other hand, if the number of produced sporozoites is high, they will be able to overcome the blockade and penetrate the salivary gland. Moreover, the mosquitoes expressing TSR-based peptides Sin-PgRTSR, Sin-PfRTSR, Sin-PfpTSR were unable to generate significant difference compared to control even if a threshold is established (Additional file 4) reinforcing the results presented by the second analysis of pBac-PgpNT line P#1.

The reduction (~30 %) of sporozoites invading salivary glands in the presence of peptides based on NH_2_-terminus of CSP from either *P. gallinaceum* or *P. falciparum* in *Ae. aegypti* is lower than that observed (60–80 %) when *A. stephensi* were injected with synthetic peptides from *P. falciparum* and infected by *P. berghei* [18]. It is likely that the amounts of recombinant peptides produced by both dsSindbis virus and the transgene were lower than the amount injected in that study, and this is reflected in the blocking efficiency like stated previously. It was reasoned that because codon optimization is crucial for heterologous expression of CSP in transgenes in *Anopheles gambiae* [38], it might also be crucial in *Ae. aegypti.* Also, CSP is not the only protein described that could promote salivary gland invasion [5, 6] and, therefore, complete inhibition may not be possible with a single class of peptides.

Recent technological advances in cellular and molecular biology, genetics and bioinformatics, have enabled the development of a transgenic *A. stephensi* transgenic lines that block the progression of the *P. falciparum* cycle rendering salivary glands free of sporozoites [39]. This transgenic line is a groundbreaking tool towards an innovative, population modification method to control vector-borne diseases. However, human malaria parasites can be transmitted by a ~465 formally recognized *Anopheles* species and ~41 are considered to be dominant vector species/species complexes, capable of transmitting malaria at a level of major concern to public health [40]. Thus, further studies on the biology of different vectors and host-pathogen interactions are needed to provide a broad spectrum of control possibilities that include all mosquitoes-parasites combinations.

## Conclusions

The interference of invasion of *Ae. aegypti* salivary gland by sporozoites achieved by endogenously-expressed peptides represents a step forward in understanding this complex stage in the parasite cycle. Together with previous studies, the data presented here indicate that peptides endogenously expressed from CSP partial NH_2_-terminus are able to compete for receptors in the salivary gland and disrupt the sporozoite/salivary gland interaction. Also, the fact that this portion of the protein from either the avian and human parasite is capable of mediating this recognition supports an evolutionarily conserved role between these parasites. The blocking effect of CSP recombinant peptides make them candidates that could be used to interfere with the parasite-salivary gland interaction and thus reduce sporozoite transmission.

## Additional files

10.1186/s12936-016-1207-8 Primers sequences used for PCR and RT-PCR.
10.1186/s12936-016-1207-8 Schematic representation of cloning into pTE/3 2 J plasmid (dsSindbis virus) and piggyBac[3Xp3-EGFP] transgenesis plasmid. The nucleotide sequence encoding recombinant peptides was inserted into pTE/3 2 J dsSindbis vector using the XbaI restriction enzyme. Abbreviations: SP6: Sindbis Promoter sequence; NSP 1, 2, 3, 4; Nonstructural viral protein; C: capsid; PE2: Envelope protein 2; Mal: *Ae. aegypti* encoding the secretory signal peptide (Mal I signal peptide); CSP-PEP: correspond to pNH2-T, RTSR and pTSR constructs; 3′UTR: dsSindbis virus untranslated region. (B) The Mal-PgpNT fragment was reamplified and inserted into pSLfa[AeVg] and flanked by *Ae. aegypti* vitellogenin promoter (AeVg) and the SV40 polyadenylation sequence (SV40). (C) The [AeVg-PgpNT-SV40] transgene was inserted into a vector plasmid composed of the coding sequences of the EGFP gene to provide a visible transformation marker driving by three copies of the Pax3 (3xP3) promoter. Arrows (LH and RH) are the terminal inverted repeats of the piggyBac transposable element.
10.1186/s12936-016-1207-8 Analysis of *Plasmodium gallinaceum* infection above the threshold (<2086 sporozoites/salivary gland) in pBac[3XP3-eGFP-AeVg-PgpNT] transgenic mosquitoes. Solid circles (wild-type) and squares (transgenic pBac-PgpNT P#1) represent individual mosquitoes with sporozoites detected in their salivary glands above the threshold of 2086 sporozoites per salivary gland. The horizontal bars represent the median. A p value of <0.05 was considered statistically significant.
10.1186/s12936-016-1207-8 Analysis of *Plasmodium gallinaceum* low levels infection in dsSindbis infected and control mosquitoes. Solid circles (control Sin-EGFP) and squares (Sin-PfpNT, Sin-PgRTSR, Sin-PfRTSR, Sin-PfpTSR) represent individual mosquitoes with sporozoites detected in their salivary glands below the threshold of 2086 sporozoites per salivary gland. The horizontal bars represent the median. A p value of <0.05 was considered statistically significant.
