# Supplementary figures and images for: Endogenously-expressed NH2-terminus of circumsporozoite protein interferes with sporozoite invasion of mosquito salivary glands
Source: Malar J. 2016 Mar 10;15:153. doi: 10.1186/s12936-016-1207-8 (PMC4785649; doi:10.1186/s12936-016-1207-8)

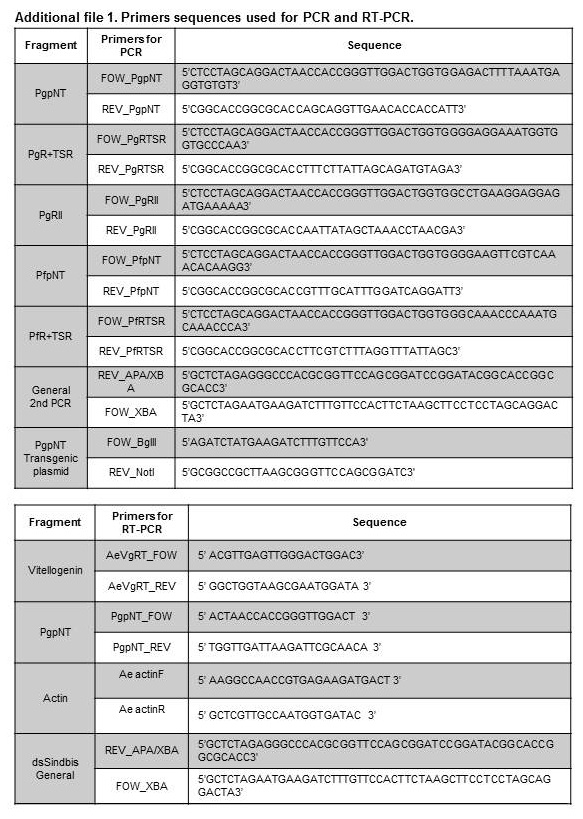

Supplement: Supplementary file 1 — 10.1186/s12936-016-1207-8 Primers sequences used for PCR and RT-PCR. [file 12936_2016_1207_MOESM1_ESM.jpg]

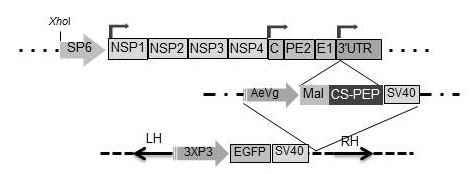

Supplement: Supplementary file 2 — 10.1186/s12936-016-1207-8 Schematic representation of cloning into pTE/3 2 J plasmid (dsSindbis virus) and piggyBac[3Xp3-EGFP] transgenesis plasmid. The nucleotide sequence encoding recombinant peptides was inserted into pTE/3 2 J dsSindbis vector using the XbaI restriction enzyme. Abbreviations: SP6: Sindbis Promoter sequence; NSP 1, 2, 3, 4; Nonstructural viral protein; C: capsid; PE2: Envelope protein 2; Mal: Ae. aegypti encoding the secretory signal peptide (Mal I signal peptide); CSP-PEP: correspond to pNH2-T, RTSR and pTSR constructs; 3′UTR: dsSindbis virus untranslated region. (B) The Mal-PgpNT fragment was reamplified and inserted into pSLfa[AeVg] and flanked by Ae. aegypti vitellogenin promoter (AeVg) and the SV40 polyadenylation sequence (SV40). (C) The [AeVg-PgpNT-SV40] transgene was inserted into a vector plasmid composed of the coding sequences of the EGFP gene to provide a visible transformation marker driving by three copies of the Pax3 (3xP3) promoter. Arrows (LH and RH) are the terminal inverted repeats of the piggyBac transposable element. [file 12936_2016_1207_MOESM2_ESM.jpg]

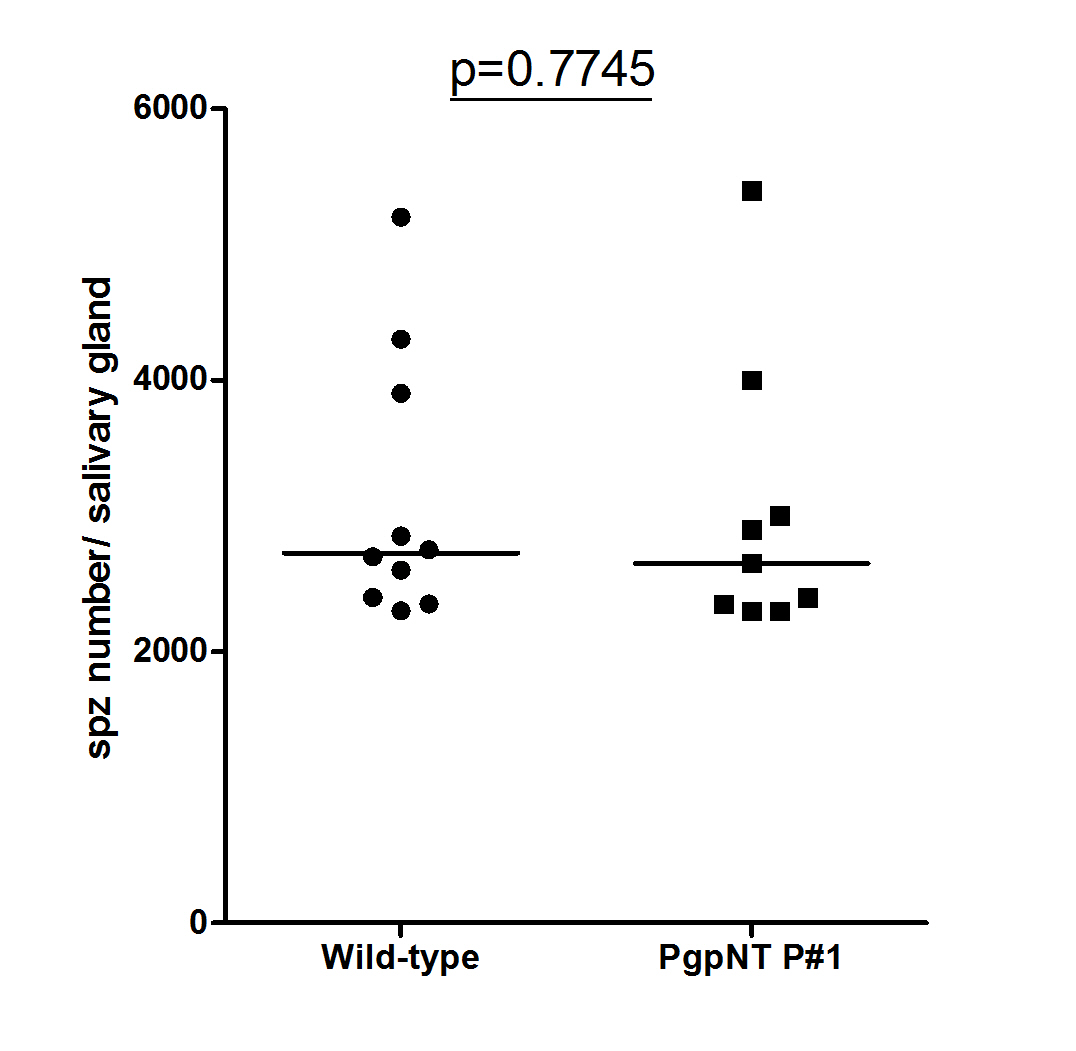

Supplement: Supplementary file 3 — 10.1186/s12936-016-1207-8 Analysis of Plasmodium gallinaceum infection above the threshold (<2086 sporozoites/salivary gland) in pBac[3XP3-eGFP-AeVg-PgpNT] transgenic mosquitoes. Solid circles (wild-type) and squares (transgenic pBac-PgpNT P#1) represent individual mosquitoes with sporozoites detected in their salivary glands above the threshold of 2086 sporozoites per salivary gland. The horizontal bars represent the median. A p value of <0.05 was considered statistically significant. [file 12936_2016_1207_MOESM3_ESM.jpg]

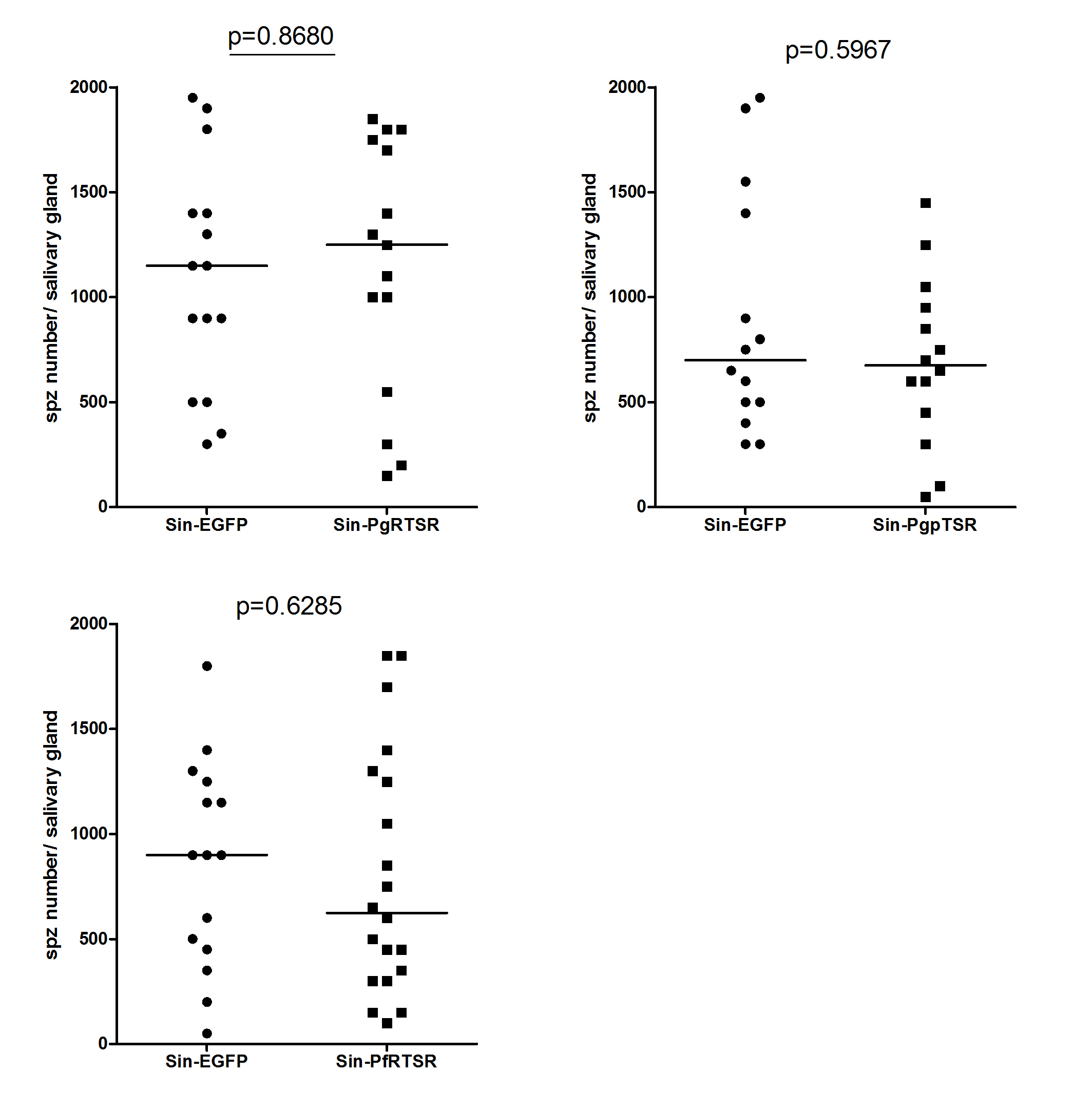

Supplement: Supplementary file 4 — 10.1186/s12936-016-1207-8 Analysis of Plasmodium gallinaceum low levels infection in dsSindbis infected and control mosquitoes. Solid circles (control Sin-EGFP) and squares (Sin-PfpNT, Sin-PgRTSR, Sin-PfRTSR, Sin-PfpTSR) represent individual mosquitoes with sporozoites detected in their salivary glands below the threshold of 2086 sporozoites per salivary gland. The horizontal bars represent the median. A p value of <0.05 was considered statistically significant. [file 12936_2016_1207_MOESM4_ESM.jpg]
